# The Eccentric Phase in Unilateral Resistance Training Enhances and Preserves the Contralateral Knee Extensors Strength Gains After Detraining in Women: A Randomized Controlled Trial

**DOI:** 10.3389/fphys.2022.788473

**Published:** 2022-03-03

**Authors:** Giuseppe Coratella, Annalisa Galas, Francesco Campa, Anna Pedrinolla, Federico Schena, Massimo Venturelli

**Affiliations:** ^1^Department of Biomedical Sciences for Health, Università degli Studi di Milano, Milano, Italy; ^2^Department of Neuroscience, Biomedicine and Movement Sciences, University of Verona, Verona, Italy; ^3^Department for Life Quality Studies, University of Bologna, Rimini, Italy; ^4^CeRISM Research Center, University of Verona, Rovereto, Italy; ^5^Department of Internal Medicine, University of Utah, Salt Lake City, UT, United States

**Keywords:** vastus lateralis, quadriceps, ultrasound, strength training, concentric, isokinetic, cross-education effect

## Abstract

The current randomized controlled study investigated whether or not the inclusion of the eccentric phase in resistance training favors the contralateral strength gains after different unilateral protocols, and whether such gains are retained after detraining. Sixty healthy women were randomly assigned to a unilateral concentric-only (CONC), eccentric-only (ECC), concentric–eccentric (TRAD) volume-equated knee extension training or control group (CON). The participants trained 2 days/week for 8 weeks and then did not train for further 8 weeks. Knee extensors isokinetic concentric, eccentric, and isometric peak torque and *vastus lateralis* muscle thickness were assessed in the contralateral limb at baseline, post-training, and post-detraining. At post-training, concentric peak torque increased in CONC [+9.2%, 95%CI (+6.2/+12.3), *p* < 0.001, ES: 0.70, 95%CI (0.01/1.39)], ECC [+11.0% (+7.7/+14.2), *p* < 0.001: ES: 0.66(0.09/1.23)] and TRAD [+8.5%(+5.7/+11.6), *p* < 0.001, ES: 0.50(0.02/0.98)]. Eccentric peak torque increased in ECC in ECC [+15.0%(+11.4/+20.7), *p* < 0.001, ES: 0.91(0.14/1.63)] and TRAD [+5.5%(+0.3/10.7), *p* = 0.013, ES: 0.50(0.05/0.95)]. Isometric peak torque increased in ECC [+11.3(+5.8/16.8), *p* < 0.001, ES: 0.52(0.10/0.94)] and TRAD [+8.6%(+3.4/+13.7), *p* < 0.001, ES: 0.55(0.14/0.96)]. No change in eccentric and isometric peak torque occurred in CONC (*p* > 0.05). Muscle thickness did not change in any group (*p* > 0.05). At post-detraining, all groups preserved the contralateral strength gains observed at post-training (*p* < 0.05). The findings showed that ECC and TRAD increased contralateral knee extensors strength in concentric, eccentric, and isometric modality, while CONC only increased concentric strength. The eccentric phase appears to amplify the cross-education effect, permitting a transfer in strength gaining toward multiple testing modalities. Both eccentric-based and traditional eccentric–concentric resistance protocols are recommended to increase the contralateral retention in strength gains after a detraining period.

## Introduction

When resistance training is systematically performed unilaterally, the training effects are also visible in the untrained limb, so that strength gains also occur in the contralateral homologous muscles (Munn et al., 2004; Manca et al., 2017). The literature refers to this phenomenon as “cross-education” or “contralateral strength training effect” (Carroll et al., 2006). The increase in contralateral strength was in first instance hypothesized to derive from neuromuscular rather than structural cross-transferred adaptations (Lee and Carroll, 2007), and thereafter shown to be mainly ascribed to increases in supraspinal neural drive (Fimland et al., 2009; Lee et al., 2009), possibly excluding any structural change in the contralateral muscle to account for the increase in strength (Carroll et al., 2006). The extent of the contralateral strength gains compared with baseline reported in two meta-analysis was *circa* +8% (Munn et al., 2004) and +12% (Manca et al., 2017), albeit similar *moderate* effect size was observed in both meta-analyses.

Unilateral dynamic traditional resistance training consists of the execution of both concentric and eccentric phase for a given exercise, and its effectiveness in promoting cross-education was already reported (Shima et al., 2002; Munn et al., 2005; Fimland et al., 2009; Pearce et al., 2013; Green and Gabriel, 2018; Leung et al., 2018). However, resistance training also includes protocols in which either the concentric-only or the eccentric-only phase is systematically performed. The literature showed that both concentric-only (Housh et al., 1996a; Weir et al., 1997) and eccentric-only (Housh et al., 1996a; Farthing and Chilibeck, 2003; Coratella et al., 2015a; Adrushko et al., 2018) unilateral training increased contralateral strength. Interestingly, when directly compared, eccentric-only vs. concentric-only training appears as more effective in promoting contralateral strength gains (Hortobágyi et al., 1997; Kidgell et al., 2015). However, when performing a direct comparison, the training–testing specificity (e.g., concentric-only training and concentric strength test) and the capacity to promote contralateral strength in non-specific testing modalities should be accounted for. The only study that has systematically compared the effects of concentric-only vs. eccentric-only training on the contralateral concentric, eccentric and isometric strength, reported greater transfer ability toward multiple strength testing modality following eccentric-only training (Kidgell et al., 2015). Although in this study the training consisted of a similar number of concentric-only or eccentric-only repetitions performed at a similar range of motion and angular velocity, each concentric-only or eccentric-only contraction was performed maximally. Because eccentric-only actions permit the exertion of supramaximal force (Fang et al., 2004), combining all factors (Coratella et al., 2019) the greater cross-education reported in the eccentric-only group may have benefited from the greater training volume. Additionally, the effects of traditional concentric–eccentric training, the most used in practice, were not concurrently examined.

When a training cessation occurs (i.e., detraining), the training-induced effects might be lost proportionally with the detraining duration (Bosquet et al., 2013). Although poorly investigated, previous studies reported that the contralateral strength gains were retained after concentric-only (Housh et al., 1996b), eccentric-only (Housh et al., 1996a), or traditional concentric–eccentric training (Shima et al., 2002; Green and Gabriel, 2018). However, these studies: (i) investigated the effects of a single unilateral strength training protocol (Housh et al., 1996a,b), (ii) did not investigate the changes in contralateral strength across multiple strength testing modalities (Shima et al., 2002; Green and Gabriel, 2018), and (iii) did not systematically examined it in women (Housh et al., 1996a,b; Shima et al., 2002). Therefore, the current study aimed: (i) to compare the cross-education effect after a unilateral volume-equated concentric-only, eccentric-only, or traditional concentric–eccentric training on the contralateral concentric, eccentric, and isometric strength and (ii) the retention of the cross-education effect after a detraining period. Additionally, contralateral changes in muscle size were also examined to possibly exclude any structural change in the contralateral muscle. It was hypothesized that the inclusion of the eccentric action could result in greater cross-education effect.

## Materials and Methods

### Study Design

The present investigation was conceived as parallel, four groups, pre–post, randomized controlled trial. Using a restricted blocked randomization (computer-generated sequence, proportion 1:1:1:1), the participants were randomized into four groups: concentric-only (CONC), eccentric-only (ECC), traditional concentric–eccentric training (TRAD), and control group (CON; Coratella and Schena, 2016). One of the researchers without any contact or knowledge of the participants completed the allocation and randomization of groups.

The sample size was calculated *a priori* using a statistical software (G-Power 3.1, Dusseldorf, Germany). Considering the study design (four groups, three repeated measures), a medium effect size *f* = 0.25, a correlation among repeated measures *r* = 0.5, a non-sphericity correction ∈ = 1, an α -error = 0.05 and a required power 1– β = 0.80, the total sample size resulted in 40 participants. To overcome any drop in statistical power due to possible dropouts, we recruited 60 participants, resulting in *a posteriori* statistical power 1– β = 0.91.

### Participants

Sixty moderately active women were recruited among a university-based population (age: 22 ± 4 years, body mass: 60.2 ± 4.3 kg, and stature: 1.64 ± 0.06 m). The participants were not engaged in any systematic resistance training for the previous 6 months. For the entire duration of the present study, the participants were not allowed to participate in any other form of resistance training activity. The overall amount of physical activity was assessed weekly for each participant using an IPA-Q questionnaire to check that no changes in the participants’ habits occurred, with pre-training values = 652(117) METs per week. Dietary intake was not monitored, but the participants were instructed not to change their usual feed behavior. People with any hip, knee, or ankle disorder, muscle injury, and users of any drug were excluded from the study. All participants signed a written informed consent which was approved by the Ethics Committee of the University of Verona and were informed that they could withdraw from the study at any time. The procedures were conducted in accordance with the international ethical standards of the Declaration of Helsinki (1975) for studies involving human subjects. The procedures were not previously registered.

### Procedures

To evaluate the knee extensors strength, isokinetic concentric, eccentric, and isometric peak torque were assessed. To evaluate possible change in muscle size, *vastus lateralis* thickness was assessed by ultrasound. All dependent parameters were assessed on the untrained limb.

The present investigation lasted a total of 19 weeks. In week-1, the participants were involved in three sessions. In the first sessions, they were familiarized with all intervention methods (CONC, ECC, and TRAD) and with the isokinetic testing modalities (concentric, eccentric, and isometric). In the second session, muscle thickness was obtained, and the participants familiarized again with all intervention methods and the isokinetic testing procedures. In the third session, the isokinetic testing procedures were assessed. From week-2 to week-9, the participants performed the intervention training. In week-10, post-training testing procedures were assessed, at least 4 days after the end of training. Then, from week-11 to week-18, the participants were involved in the detraining period and were instructed not to train. Lastly, at week-19, the post-detraining testing procedures were assessed. Each testing assessment was performed by the same experienced operator.

### Isokinetic Test

An isokinetic dynamometer (Cybex Norm, Lumex, Ronkonkoma, United States) was used to measure the knee extensors strength. The procedures followed previous protocols (Coratella et al., 2015a,b). Briefly, the device was calibrated according to the manufacturer’s recommendations and the center of rotation was aligned with the tested knee. The participants were seated on the dynamometer’s chair, with their trunks slightly reclined backward and a hip angle of 85°. Two seatbelts secured the trunk and one strap secured the tested limb, while the untested limb was secured by an additional lever. The testing measurements were preceded by a standardized warm-up, consisting of three sets × 10 repetitions of weight-free squats (Coratella et al., 2018). Knee extensor strength was measured in concentric (1.05 deg. s^−1^), eccentric (−1.05 deg. s^−1^), and isometric (60 deg., 3 s) modalities (Coratella et al., 2015a,b). Each testing modality consisted of three maximal trials and was separated by 2 min of passive recovery. Strong standardized encouragements were provided to the participants to maximally perform each trial throughout the whole test, for each repetition performed.

### Muscle Thickness

Vastus lateralis thickness was assessed *in vivo* at rest in VL and GM by B-mode ultrasound (LOGIQS7, GE©, Fairfield, Connecticut, United States) with a 5-cm linear-array probe (mod. 9 l, 3.1–10.0 MHz). The participants lay supine on the examination bed with the hip joint extended and the knee joint almost fully extended (170° extension, with 180° full extension). The probe was held perpendicular to the skin surface by an expert operator, which ensured minimal pressure was applied to the muscle belly examined. No visually identifiable muscle compression was detected on the scan, as checked real time during the scan acquisition (Coratella et al., 2020). A transmission gel was applied to improve acoustic coupling. Images were obtained along the vastus lateralis mid-sagittal plane, which included both superficial and deep aponeuroses, and the probe was oriented so that a number of clearly visible fascicles were captured. Careful manipulation was provided to align the transducer to the muscle fascicle plane and optimize the echogenicity of muscle fascicles (Coratella et al., 2020). The 50% of vastus lateralis length and width. Two images were recorded. The images were analyzed offline using an open source computer program (ImageJ 1.44b, National Institutes of Health, United States). Muscle thickness was defined as the distance between the superficial and deep aponeurosis and averaged across three measurements (Coratella et al., 2015a,b).

### Intervention

The intervention was previously used to examine the changes in the trained limb (Coratella et al., 2021). The intervention lasted 8 weeks. In the first week, the participants performed one training session, since ECC would possibly have resulted in muscle damage (Coratella and Bertinato, 2015), while from the second week on they performed two training sessions per week, for a total of 15 sessions. The unilateral dynamic constant external load knee extension training was performed on a gym device (Leg extension Technogym, Cesena, Italy). Following previous recommendations to equalize training volume (Coratella et al., 2019), we manipulated the number of repetitions (sets × repetitions), the load considered as %1-RM, fixing the consistent within-subject load angular displacement (approximately 85 deg.; Coratella et al., 2015b) and the time under tension (1.5 s; Coratella et al., 2015b) for each phase (concentric or eccentric). Visual feedback (time = 1.5 s) was provided to the participants to maintain the required time under tension (Coratella et al., 2015a,b). Therefore, for each training session, CONC performed six sets × seven repetitions at 85%1-RM; ECC performed five sets × six repetitions at 120%1-RM; TRAD performed four sets × five repetitions at 90%1-RM, while CON did not train (Coratella and Schena, 2016). Knee extensors 1-RM was performed on the same device used for the training (Leg extension Technogym, Cesena, Italy), in line with previous procedures (Coratella and Bertinato, 2015; Coratella et al., 2015a,b). During each repetition performed in CONC, an operator lowered the lever to relieve each participant from the eccentric phase; during each repetition performed in ECC, an operators lifted the lever to relieve each participant from the concentric phase; each repetition in TRAD was performed autonomously by the participants without the help of any operator (Coratella and Schena, 2016). The intervention was performed on the dominant limb. The participants were instructed to relax the untrained limb as much as possible. Each set was separated by 3 min of passive recovery. Each session was separated by at least 3 days. After the post-training testing session, the participants did not train for 8 weeks.

### Statistical Analysis

The statistical analysis was performed using a statistical software (SPSS 26.0, IBM, Armonk NY, United States). The normality of data was checked using the Kolmogorov–Smirnov test, and all data were found to be normal. The test–retest reliability was measured using an intraclass correlation coefficient (ICC) and interpreted as follows: *α* ≥ 0.9 = excellent; 0.9 > α ≥ 0.8 = good; 0.8 > α ≥ 0.7 = acceptable; 0.7 > α ≥ 0.6 = questionable; 0.6 > α ≥ 0.5 = poor (Tavakol and Dennick, 2011). The post-training and post-detraining changes in contralateral strength in the intervention groups were calculated in accordance with previous recommendations to account for the changes in CON (Carroll et al., 2006):


$$\mathrm{change}=\left[\left(T\;\mathrm{Post}–T\;\mathrm{pre}\right)/T\;\mathrm{Pre}\right]–\left[\left(\mathrm{CON}\;\mathrm{Post}–\mathrm{CON}\;\mathrm{pre}\right)/\mathrm{CON}\;\mathrm{pre}\right]$$

where T indicates one out of the intervention group.

To check the within- and between-group difference in isokinetic concentric, eccentric and isometric peak torque and *vastus lateralis* muscle thickness mixed-factor model was separately performed for each dependent parameter. Additionally, to calculate the between-group (four groups: CONC, ECC, TRAD, and CON) differences in temporal adaptations (three times: pre, post-training, and post-detraining), data were log-transformed and analyzed using an ANCOVA, considering pre values as covariate. Multiple comparisons were calculated using Bonferroni’s correction. Significance was set at *α* < 0.05. Data are reported as mean with SD. Changes are reported as % change with 95%CI and Cohen’s *d* effect size (ES) with 95%CI. ES was interpreted as follows (Hopkins et al., 2009): 0.00–0.19: *trivial*; 0.20–0.59: *small*; 0.60–1.19: *moderate*; 1.20–1.99: *large*; ≥ 2.00: *very large*.

## Results

No injury was occurred during the whole duration of the study. The overall rate of compliance to the training program was 96.1% for CONC, 92.3% for ECC, and 93.7% for TRAD. The test–retest reliability was *excellent* for concentric (ICC = 0.932), eccentric (ICC = 0.910) and isometric (ICC = 0.928) peak torque and for *vastus lateralis* muscle thickness (ICC = 0.901). The standard error of the measurement was 6.3 Nm for the concentric, 9.9 Nm for the eccentric, 9.3 N for the isometric peak torque and 0.9 mm for muscle thickness. CONC, ECC, TRAD, and CON did not show any between-group difference at baseline.

**Figure 1:**
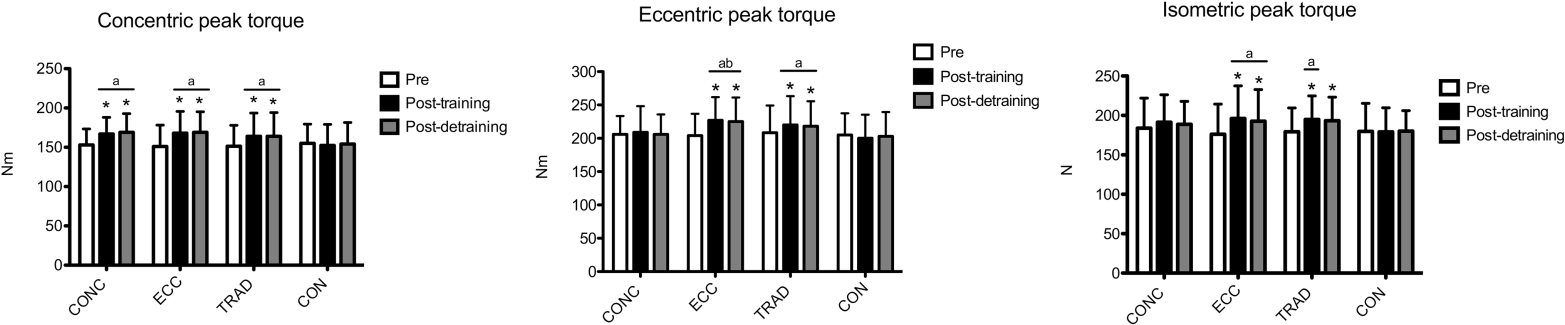
The changes in contralateral concentric, eccentric, and isometric peak torque are shown for the concentric-only (CONC), eccentric-only (ECC), traditional concentric–eccentric (TRAD) volume-equated training groups and control (CON). Concentric peak torque similarly increased in all training groups more than CON and was similarly retained after the detraining period. Eccentric peak torque increased and was retained similarly in ECC and TRAD more than CON, but only ECC was different from CONC. Isometric peak torque increased similarly in ECC and TRAD more than CON, but TRAD did not differ from CON at post-detraining. *p < 0.05 vs. Pre; ^a^*p* < 0.05 vs. CON; and ^b^*p* < 0.05 vs. CONC.

The results for concentric peak torque are shown in Figure 1. Time × group interaction (*p* < 0.001) was found for concentric peak torque. Compared to pre, within-group analysis showed that concentric peak torque increased at post-training in CONC [+9.2%, 95%CI (+6.2/+12.3), *p* < 0.001, ES: 0.70, 95%CI (0.01/1.39)], ECC [+11.0%(+7.7/+14.2), *p* < 0.001: ES: 0.66(0.09/1.23)] and TRAD [+8.5%(+5.7/+11.6), *p* < 0.001, ES: 0.50(0.02/0.98)], while CON did not show any change (*p* > 0.05). Between-group analysis showed no difference between the intervention groups, whose increases were greater than CON (*p* < 0.05). At post-detraining, concentric peak torque was still greater compared to pre in CONC [+9.9%(+6.2/+13.7), *p* < 0.001, ES: 0.69(0.07/1.31)], ECC [+11.6%(+7.6/+15.6), *p* < 0.001, ES: 0.66(0.02/1.30)] and TRAD [+8.1%(+4.3/+11.8), *p* < 0.001, ES: 0.43(0.00/0.86)]. Between-group analysis showed no difference between the intervention groups, whose increases were greater than CON (*p* < 0.05).

The results for eccentric peak torque are shown in Figure 1. Time × group interaction (*p* < 0.001) was found for eccentric peak torque. Compared to pre, within-group analysis showed that eccentric peak torque increased at post-training in ECC [+15.0%(+11.4/+20.7), *p* < 0.001, ES: 0.91(0.14/1.63)] and TRAD [+5.5%(+0.3/10.7), *p* = 0.013, ES: 0.50(0.05/0.95)], while no change occurred in CONC and CON (*p* > 0.05). Between-group analysis showed that ECC and TRAD had similar increases that were greater than CON (*p* < 0.05), but only ECC was greater than CONC [+10.7%(+3.1/18.0), *p* = 0.004, ES: 0.75(0.26/1.25)]. At post-detraining, eccentric peak torque was still greater compared to pre in ECC [+10.3%(+5.4/+15.1), *p* < 0.001, ES: 0.61(0.11/1.11)] and TRAD [+4.7%(+ 0.1/+9.3), *p* = 0.047, ES: 0.39(0.00/ 0.78)]. Between-group analysis showed that ECC and TRAD had similar retentions, but only ECC differed from CONC and CON (*p* < 0.05), while TRAD only from CON (*p* < 0.05).

The results for isometric peak torque are shown in Figure 1. Time × group interaction (*p* = 0.011) was found for isometric peak torque. Compared to pre, within-group analysis showed that isometric peak torque increased at post-training in ECC [+11.3(+5.8/16.8), *p* < 0.001, ES: 0.52(0.10/0.94)] and TRAD [+8.6%(+3.4/+13.7), *p* < 0.001, ES: 0.55(0.14/0.96)], while no change occurred in CONC and CON (*p* > 0.05). Between-group analysis showed that ECC and TRAD had similar increases that were greater than CON (*p* < 0.05) but not CONC (*p* > 0.05). At post-detraining, isometric peak torque was still greater in ECC [+9.4%(+2.7/16.0), *p* = 0.003, ES: 0.42(0.00/0.84)] and TRAD [+7.8%(+1.4/14.1), *p* = 0.011, ES: 0.47(0.04/0.90)]. Between-group analysis showed that ECC and TRAD had similar retentions, but only ECC differed from CON (*p* < 0.05).

The baseline values for vastus lateralis muscle thickness were 20.6(3.2) mm for CONC, 21.3(2.9) mm for ECC, 20.6(3.6) mm for TRAD, and 20.9(2.6) mm for CONC. No time × group interaction (*p* = 0.887) was found. Compared to pre, within-group analysis did not show any change at post-training and post-detraining in any group.

## Discussion

The current study was designed to investigate (i) the contralateral effects of unilateral volume-equated concentric-only, eccentric-only, and concentric–eccentric knee extension training on the knee extensors concentric, eccentric, and isometric peak torque and *vastus lateralis* muscle thickness and (ii) the contralateral muscle strength and size retention after a detraining period. The findings showed that ECC and TRAD increased contralateral knee extensors strength in concentric, eccentric, and isometric modality, while CONC only increased concentric strength. All post-training strength gains were retained after the 8-week detraining period. No change in contralateral *vastus lateralis* thickness was observed in any intervention group. Remarkably, the inclusion of a control group and at least two familiarization sessions were strongly advocated to decrease the risk of bias and possibly catch the “actual” cross-education (Munn et al., 2004; Carroll et al., 2006; Manca et al., 2017). As hypothesized, the systemic inclusion of the eccentric phase in resistance training seems to enhance the cross-education effect.

### Post-training Adaptations

A major finding of the present study was that both ECC and TRAD increased contralateral strength across multiple testing modalities, while CONC only increased contralateral concentric peak torque. In a previous study that did not include traditional concentric–eccentric training, it was reported that eccentric-only training increased the concentric, eccentric, and isometric peak torque, while concentric-only training increased only the concentric peak torque (Kidgell et al., 2015). Another study examined the effects of unilateral eccentric-only training, similarly showing gains in contralateral concentric, eccentric, and isometric peak torque (Adrushko et al., 2018). Using different study design, eccentric-only training increased eccentric and isometric peak torque more than the gains in concentric and isometric peak torque observed after concentric-only training (Hortobágyi et al., 1997). In contrast, changes in contralateral eccentric but not concentric peak torque were also reported after eccentric-only training (Farthing and Chilibeck, 2003;Seger and Thorstensson, 2005; Lepley and Palmieri-Smith, 2014). Additionally, the different isokinetic unilateral training modality (Seger and Thorstensson, 2005; Lepley and Palmieri-Smith, 2014) may have resulted in a poor transfer in concentric peak torque. Possibly in line, the greater extent of the increase in eccentric vs. concentric and isometric peak torque in ECC is consistent with the training–testing specificity principle, as also previously shown (Coratella et al., 2015a). Traditional unilateral concentric–eccentric training was shown to improve dynamic 1-RM (Pearce et al., 2013), concentric (Seger and Thorstensson, 2005), and isometric peak torque (Shima et al., 2002; Pearce et al., 2013; Green and Gabriel, 2018; Leung et al., 2018; Chaouachi et al., 2019), while it appears that no study has assessed eccentric peak torque. Concerning the concentric-only training-induced cross-education effect, the literature is inconsistent. Gains in contralateral 1-RM (Housh et al., 1996b; Weir et al., 1997) but not concentric peak torque (Housh et al., 1996a) or increments in concentric (Kidgell et al., 2015) or isometric peak torque (Zult et al., 2016) were previously shown. Overall, the *moderate* contralateral strength gains extent observed here was consistent with what reported in different meta-analysis (Munn et al., 2004; Cirer-Sastre et al., 2017; Manca et al., 2017). Although in line with the literature, the cross-education extent could possibly be associated with the training load, so that the current high-load training (>85% 1-RM) might have enhanced the contralateral strength gains (Cirer-Sastre et al., 2017). Additionally, the direction of the cross-education from the dominant to the non-dominant limb may have also contributed to develop the contralateral strength increases (Farthing et al., 2005). Lastly, it was shown that the cross-education effect is greater in lower vs. upper limb (Manca et al., 2017), so the elbow flexors training (Farthing and Chilibeck, 2003) may have not maximized the contralateral effects.

No change in contralateral *vastus lateralis* muscle thickness was observed in any group. This is in line with the literature, since the studies that have examined the contralateral structural changes following unilateral resistance training, did not observe any change in muscle thickness (Farthing and Chilibeck, 2003; Farthing et al., 2005; Coratella et al., 2015a; Kidgell et al., 2015). Therefore, the present results support that the cross-education effect in resistance training is mediated by neural mechanisms only.

The mechanisms underneath the cross-education effect have been summarized in a previous review, that highlighted the role of the ipsilateral primary motor cortex in modulating the cross neural drive (Ruddy and Carson, 2013). However, the present design did not permit examining further any mechanism. Using procedures that allowed deepening the mechanistic explanations, it was reported that following eccentric-only vs. concentric-only training, corticospinal excitability increased more during the eccentric peak torque, with no change observed during the concentric peak torque (Kidgell et al., 2015). Additionally, corticospinal and intra-cortical inhibition were overall reduced following eccentric-only but not concentric-only training especially during the isometric peak torque (Kidgell et al., 2015). Interestingly, performing maximal eccentric actions was also shown to increase the activity of the central nervous system, (Fang et al., 2004), so it is plausible that greater inter-hemispheric stimuli occurred (Ruddy and Carson, 2013). As a whole, the inclusion of the eccentric phase in both ECC and TRAD may have induced a series of favorable neural adaptations that allowed increasing contralateral strength when tested in multiple modalities.

### Post-detraining Adaptations

Another major finding is that each training modality retained the contralateral strength gains after an 8-week detraining period. No study has investigated concurrently the cross-education retention after different resistance training and multiple strength testing modalities, so a direct comparison with the literature is challenging. Indeed, the previous studies examined the retention in contralateral strength gains after single resistance training protocols. Unilateral eccentric-only training was shown to retain contralateral increase in eccentric 1-RM (Housh et al., 1996a). Traditional concentric–eccentric training was inconsistently shown to retain (Green and Gabriel, 2018; Chaouachi et al., 2019) or not retain isometric peak torque (Shima et al., 2002) after similar training volume, albeit performed on different muscles, possibly explaining the different results. Moreover, traditional training also retained the concentric 1-RM after 4 weeks (Chaouachi et al., 2019). Concentric-only training retained the contralateral strength increments in concentric 1-RM (Housh et al., 1996b; Weir et al., 1997). The maintenance of the strength increases in the contralateral limb seems to be associated with a retention of the neuromuscular central adaptations induced by the unilateral training of the opposite limb (Green and Gabriel, 2018), even though a previous study did not observe such a retention (Shima et al., 2002). Further studies are required to elucidate this point and verify the effect of concurrent different training regimens on the neuromuscular adaptations.

It is acknowledged that the current study presents some limitations. The currents results are related to the volume, the muscle group, and the exercise performed here, and it is possible that different combinations of this factor may results in different outcomes. Moreover, different duration of both the training and detraining period could possibly have repercussion on the dependent parameters. Indeed, no mechanistic explanation was provided, and it is acknowledged that assessing neuromuscular variables may enrich the state of art of the cross-education effect.

In conclusion, both ECC and TRAD promoted and retained contralateral strength gains in concentric, eccentric, and isometric peak torque, while CONC only increased and maintained contralateral concentric peak torque in women. Performing systemically the eccentric phase during unilateral resistance training appears beneficial to induce and retain the strength increases in the contralateral untrained limb. Both in sports practice and rehabilitation, the current findings may be helpful to maximize the effects of unilateral resistance training when exercising bilaterally is not possible. For example, in case of an immobilized limb due to an injury, the rehabilitation process may start with a contralateral training without the need to wait for a mobility recovery in the injured limb. This may fasten the recovery, and should the training being performed including the eccentric phase, this would maximize the contralateral strength transfer and retention. In some sports practice (e.g., racquet sports or throwing), many exercises are performed unilaterally by the dominant limb because of the demands of the sport. Notwithstanding, the contralateral muscles would benefit from an eccentric-based training, so to increase its strength, saving time for more technical drills. The strength adaptations would remain in case of a training interruption, for example, off season, so to facilitate the pre-season training. However, it should be remarked that the contralateral effect does not include any structural change, and this must be obtained by means of a focused training.

## Data Availability Statement

The datasets presented in this study can be found in online repositories. The names of the repository/repositories and accession number(s) can be found at: https://zenodo.org/record/5545794#.YVhJAkZByu4.

## Ethics Statement

The studies involving human participants were reviewed and approved by University of Verona. The patients/participants provided their written informed consent to participate in this study.

## Author Contributions

GC, FS, and MV conceived the study and wrote the draft. GC, AG, FC, and AP collected and analyzed the data. All authors reviewed and agreed with the final version of the manuscript.

## Conflict of Interest

The authors declare that the research was conducted in the absence of any commercial or financial relationships that could be construed as a potential conflict of interest.

## Publisher’s Note

All claims expressed in this article are solely those of the authors and do not necessarily represent those of their affiliated organizations, or those of the publisher, the editors and the reviewers. Any product that may be evaluated in this article, or claim that may be made by its manufacturer, is not guaranteed or endorsed by the publisher.
